# A Novel Pathway for Integrating Ethics into Digital Technology Design: A Person-Centred Co-Design Approach Developed in the Context of Assistive Technologies for Older Adults

**DOI:** 10.1007/s11948-026-00584-1

**Published:** 2026-03-03

**Authors:** Tania Moerenhout, Inga Hunter, Angela Ballantyne

**Affiliations:** 1 Department of Bioethics, University of Otago, PO Box 56, Dunedin, 9054 Aotearoa New Zealand; 2 School of Management and Marketing, Massey University, Palmerston North, Aotearoa New Zealand; 3 Department of Primary Health Care & General Practice, Wellington, and Department of Bioethics, University of Otago, Dunedin, Aotearoa New Zealand

**Keywords:** Integrated ethics, Embedded ethics, Health technology, Digital assistive technologies, Older adults

## Abstract

How can we effectively incorporate ethical considerations into technology design? Several frameworks and evaluation methods focused on ethical aspects of new and emerging technologies have been proposed over the past two decades. Despite this, we still lack a robust pathway for integrating ethical considerations into the design process. We examine three existing methods: ethical technology assessment, value-sensitive design, and embedded ethics. Drawing on their strengths and limitations, we propose a novel pathway for integrating ethics (PIE) that is flexible, multi-disciplinary, and person-centred. It consists of four stages: (1) bringing ethicists and designers together; (2) identifying ethical tensions; (3) exploring solutions to ethical tensions through stakeholder engagement; (4) integrating identified solutions into technology design. This pathway has been developed in the context of digital assistive technology for older adults’ care, building on the principles of person-centred care and co-design, and embeds four fundamental values: inclusivity, collaboration, flexibility, and practicality. This novel approach could also be expanded into other areas of digital health technology design and development.

## Additional Instruction Abstract

The global aging population faces significant health and well-being challenges. Many older adults prefer to age in their own homes and community. Digital assistive technologies, such as sensors, wearables, and smart devices, offer promising solutions to support independent living while reducing burdens on caregivers and healthcare professionals. However, ethical concerns—including privacy, trust, ageism, loss of self-determination, and equitable access—limit the uptake and sustained use of digital assistive technologies. To address these challenges, we propose an integrated ethics approach grounded in two fundamental principles: person-centred care and co-design. These principles foster inclusivity, flexibility, collaboration, and practicality, forming a robust ethical foundation for the use of assistive technology solutions. Our ‘pathway to integrating ethics’ approach enables structured engagement between ethicists and technology developers, ensuring ethical considerations are embedded throughout the design process rather than treated as they occur after release. Key components include (1) a *close collaboration* between the design team and ethicists throughout the project, within clearly defined terms and conditions, (2) a strong emphasis on *stakeholder involvement* across diverse user groups, and (3) *proactive identification and resolution of ethical tensions* that arise in practice.

## Introduction

In this paper, we propose a novel approach to integrating ethical considerations into technology design in a way that is flexible, multi-disciplinary, and person-centred. This approach is called the Pathway for Integrating Ethics (PIE). While the primary aim of the paper is to propose PIE as a method for incorporating ethics into technology design, the approach requires an in-depth understanding of the context, interests, tensions, and perspectives of the involved stakeholders. As a case study to demonstrate the application of PIE, we will focus on the role of digital assistive technologies (DATs) in the context of care for older adults. Digital assistive technologies provide a relevant tool for the development and application of PIE because they present complex ethical tensions that need to be adequately addressed prior to successful uptake within communities (Felber et al., 2023; Sundgren et al., 2020; Wang et al., 2023; Wangmo et al., 2019; Zhu et al., 2022).

DATs offer the potential to improve the quality of life and reduce the socio-economic burdens of a rapidly aging population (Chabot et al., 2019). They come in many forms: smart home applications, fall detection sensors, wearables, and robotics (Byrne et al., 2018). However, various ethical challenges arising from introducing these technologies remain unsettled. Developing technology for older adults, which allows them to age in place, raises ethical questions of how to balance their safety, autonomy, and quality of life while managing the care responsibilities for family and health systems (Berridge, 2016; Grosen & Hansen, 2021). The introduction of assistive technologies involves complex trade-offs between ethical values (e.g., safety versus privacy) and tensions between the interests of different stakeholders (e.g., older adults and their children) (Berridge et al., 2020; Grosen & Hansen, 2021; Pol et al., 2016; Street et al., 2022). If we do not address these tensions, we risk developing technology for aging-in-place that is biased, unethical, or simply rejected. First, we present an illustrative scenario to exemplify the ethical issues that may arise.

### Illustrative Scenario

Helena is 81 years old and lives by herself in a small cottage about a fifteen-minute drive from the town center. She has two neighbors who are also in their eighties, and there are a few young families living a bit further down the same street. Two months ago, Helena ended up in hospital after a fall. Investigations showed she has developed neuropathy in both lower legs due to longstanding type 2 diabetes. Helena has two children who do not live nearby, and both are worried about her well-being. The children have organized biweekly visits among them but feel they cannot keep this up. They are adamant that she needs monitoring in the house, and her tech-savvy son asks her to install a video camera in the living room as well as to use a smart pill box to check her medication intake so she can be reminded when she forgets. He has just seen how a friend’s parents installed a smart home voice assistant and use it to dim their lights or ring their family and friends and wonders if Helena would like this too. They also encourage her to wear a smartwatch whenever she goes out, so they can track her if something happens. Helena does not want any technology monitoring her in the house. She has not told her children that she has a romantic relationship with one of her neighbors, and she is certain they would not approve (Berridge et al., 2020). She often skips her morning dose of diabetes medication if she feels unwell and fears the children would make a fuss if they noticed this with the smart pillbox use. Helena has grown quite fond of the biweekly visits over the past couple of months and hopes the children will keep these going. When Helena declines any technology use but does not give a proper explanation, her children become angry with her and give her the choice between accepting the monitoring systems or moving into residential care.

**Table:** 

In the following sections, we briefly describe the context of assistive technology use insupporting older adults to age in place. We then explore existing methods for integratingethics into technology design and present our pathway for integrating ethics (PIE).	

### Aging-In-Place

Supporting the health and well-being of older adults is a global health priority affecting both high- and low- to middle-income countries (World Health Organisation, 2022). Many older adults prefer to age in their own homes and community (Ouden et al., 2021; Wiles et al., 2012). Aging-in-place is defined as “the ability to live safely, independently, and comfortably in one’s own home and community, regardless of age, income, or competence level.”(CDC, 2009; Pani-Harreman et al., 2021). Aging-in-place may improve quality of life and, at the same time, reduce the socio-economic burden of a rapidly aging population (Ollevier et al., 2020; Wiles et al., 2012).

However, aging-in-place often requires the presence of informal caregivers to make it possible. This does not only mean caregivers need to reside nearby, but they also need to be able to dedicate the time and work necessary for the older person’s care, which can be a demanding role to fulfil (Lindt et al., 2020; Quinlan et al., 2022). DATs may provide some respite for caregivers by reducing stress, providing convenience, and saving time (Albers et al., 2022) They may also reduce the need for caregivers to live nearby or to organise frequent visits, as described in the scenario above, thus reducing the risk of caregiver burnout (Lindt et al., 2020).

### Digital Assistive Technology

DATs can play a central role in increasing the ability of older adults to age in their homes and communities (Chabot et al., 2019). DATs, i.e., sensors, wearables, and smart devices to monitor, assess, and support older adults, have been heralded as a transformative solution to support aging-in-place while limiting burdens on caregivers and healthcare professionals (Hunter et al., 2021; Stavropoulos et al., 2020). DAT comprises four main categories: smart home applications (usually assisting with activities of daily living (ADL) and general health monitoring), intelligent life assistants (such as fall detection and home control systems), wearables, and robotics (Byrne et al., 2018). These increasingly integrate artificial intelligence (AI). An evidence base regarding the safety and efficacy of DAT is in development: there seems to be a positive effect on chronic disease management, safety, physical functioning, and depression, but the impact on hospital admissions, quality of life, and cognitive functioning is unclear (Chabot et al., 2019; Chan et al., 2021; Liu et al., 2019; Moyle et al., 2021). Evidence for caregiver relief is also mixed: some studies showed that DATs may decrease caregiver burden, while one specifically looking at people with dementia did not show a clear effect on caregiver outcomes (Gaugler et al., 2021; Wang et al., 2024b).

Adoption rates for smart home technology are currently low (Pirzada et al., 2022; Wang et al., 2023). Whilst the novelty of such technologies, along with technical and usability issues, all play a role, ethical challenges such as concerns related to privacy, trustworthiness, ageism, loss of self-determination, and equity of access also limit uptake and sustained use of DAT (Lämås et al., 2020; Pirzada et al., 2022; Stypinska, 2022; Sundgren et al., 2020; Wang et al., 2023). For example, despite their long-time availability, emergency alarm systems (e.g. neck pendants) have proven contentious among older adults, with high drop-out in their use – they often end up in drawers rather than being worn due to a fear of negative stereotyping and ageism (Elers et al., 2018; Heide, 2022). Many of these ethical issues arising with DAT remain unsettled (Felber et al., 2023; Ho, 2020; Sundgren et al., 2020; Wang et al., 2023; Wangmo et al., 2019; Zhu et al., 2022), such as tensions between safety and privacy (Berridge, 2016; Grosen & Hansen, 2021; Pol et al., 2016; Street et al., 2022), how DAT may affect loneliness and social isolation (Barbosa Neves et al., 2021; Latikka et al., 2021), and the potential disagreement of older adults and their families on DAT use (Berridge et al., 2020). These ethical tensions are also captured in our scenario as described above. This is further complicated by AI integration, where additional questions of data quality management, distributive justice, and human contact are raised as ethical priorities (Wangmo et al., 2019). Although DAT use presents significant potential benefits to support older adults with aging-in-place and aging well, substantive ethical issues such as those described above must be addressed.

### Ethical Technology for Older Adults

We suggest that two principles are essential to the ethical delivery of health services for older adults: person-centred care and co-design.

*Person-centred care* offers a holistic approach to dealing with multimorbidity and chronic illness, requiring multidisciplinary and interprofessional care, support, and coordination. The Institute of Medicine puts forward the most widely accepted definition of person-centred care: “care that is respectful of and responsive to individual patient preferences, needs, and values and ensuring that patient values guide all clinical decisions”(Institute of Medicine (US), 2001). A person-centred care approach to health requires attention to the different values and priorities of different social and cultural groups. For example, connections to person, place, and culture are particularly central to the well-being of Indigenous people (Quigley et al., 2022; Wiles et al., 2012). Identifying specific needs and values requires engagement with users from many different backgrounds to truly capture these needs and values. Rather than treating patient involvement as a passive process, a more active, collaborative process has been suggested, where patients and other stakeholder become true partners (Meskó & de Bronkart, 2022). From a technology perspective, this can be translated into the values of inclusivity and flexibility, which we will discuss below.

Co-creation, co-design, and co-production have all been used to describe the participation and involvement of stakeholders in public health initiatives and innovation (Lipton et al., 2025; Sanz et al., 2021; Vargas et al., 2022, 2025). Here, we will focus on *co-design*, understood as the active collaboration between stakeholders in designing solutions to prespecified problems, promoting stakeholder participation to formulate or improve specific concerns (Vargas et al., 2022). Stakeholder engagement can reduce waste in health research by reducing misalignment between researchers’ aims and the needs of end-users (Slattery et al., 2020). Health research co-design has shown mutual benefit to participating patients and researchers (Slattery et al., 2020). Similarly, co-design has the potential to improve the outputs of DAT development. Here, co-design takes place at two different levels. First, by involving end-users in the design process, as discussed above (Sanz et al., 2021). Second, by establishing a collaboration between technology designers and bioethicists aimed at identifying and resolving ethical tensions that may arise with technology use, as described in our scenario. Within our context, this implies not only the value of collaboration but also practicality. When technology developers, bioethicists, and end-users come together in a co-design process, there will undoubtedly be trade-offs. It may mean not all of the end-users requirements can be realised with the available resources or that not all of the ethical tensions can be fully resolved. As co-design processes are embedded in real-life circumstances and their inevitable messiness, it requires a pragmatic approach to finding ways forward. Adhering to *person-centred care* and *co-design principles* in health technology development leads to the four values of *inclusivity*,* flexibility*,* collaboration*,* and practicality*, which will lay the foundation for our novel integrated ethics approach.

## Toward Ethical, Person-Centred Design: Existing Approaches

Several methods have been developed over the past two to three decades to evaluate emerging health technologies from an ethical perspective. A recent review study found that most of these ethical frameworks aim to descriptively screen or normatively evaluate health technologies, or they do both (Vandemeulebroucke et al., 2022). The review identified three major background contexts in which these frameworks operate: health technology assessment, value-sensitive design, and responsible research and innovation. Given these backgrounds, we have selected three aligning ethical frameworks to consider: ethical technology assessment (ETA), value-sensitive design (VSD), and embedded ethics. Although there are points of distinction, embedded ethics shares key commonalities with responsible research and innovation, offering a more practical method for ethicists and developers to collaborate on addressing ethical issues (McLennan et al., 2022). By focusing on these three methods, we ensure representation of both theory-grounded and theory-flexible approaches, as well as assessment and accompaniment techniques. Within the typology of ethics methods in design processes, ETA falls under theory-grounded assessment approaches, VSD represents a theory-grounded accompaniment method, and embedded ethics aligns with theoretically-flexible accompaniment techniques, which could also contain some level of assessment (Ozkaramanli et al., 2024). All three methods present strengths and weaknesses, and no single approach has emerged as a preferred method, leading to unresolved questions about their implementation (Vandemeulebroucke et al., 2022). In the next section, we consider these three approaches to ethical technology design and assessment, explaining their advantages and limitations.

### Ethical Technology Assessment

ETA is a qualitative ‘checklist’ consisting of nine ethical domains with which new technologies can be evaluated (Palm & Hansson, 2006). The checklist was not designed as a ‘tick-the-box’ exercise: the domains provide themes and values to elicit critical reflection on the impact of the technology under review. Rather, it was meant to be undertaken in dialogue with technology developers, not at one point in time, but in a continuous process (Palm & Hansson, 2006). In recent years, however, the ETA framework has also been used as a foundation to create decision-making checklists for health technologies, for example, in digital health research (Nebeker et al., 2021). The ETA framework has been criticized pointing out three main limitations: (1) there is an emphasis on adverse effects, thereby possibly neglecting potential benefits; (2) using predetermined ethical domains may mean specific challenges of new technologies are missed; (3) the framework does not give attention to diversity and local and cultural settings (Kiran et al., 2015). All of the above leads to the question of whether we should look beyond checklists or predetermined values (Kiran et al., 2015).

In addition, the framework would require an ethics expert to examine the new technology in light of the nine domains. ETA does not clarify how this collaboration could be constructed. Two problems arise from this. The first is a potential conflict of interest any involved bioethicists may have, in particular when they are paid by the industry partner to do this work. The second is the risk of tokenism, where the involved bioethicists may not have decision-making power and may not be able to stop or change ethically questionable choices, but the process is deemed ‘ethical’ because of their involvement. This is also sometimes referred to as ‘ethics washing’ (Hao, 2019). Lastly, ETA does not offer guidance on stakeholder involvement in the assessment. This means ETA may struggle to be inclusive and provide a truly person-centred co-design approach. Ethics by Design, a similar approach that has been suggested more recently for AI applications, encounters similar issues as it uses predetermined values and does not incorporate stakeholder engagement (Brey & Dainow, 2023). Ethical tensions such as those identified in our illustrative scenario, i.e., the burden on informal carers, the risk of social isolation, and the disagreement between older adults and their families, may be missed without inclusivity as a central value.

### Value-Sensitive Design

Value-sensitive design (VSD) introduces a second method for ethical technology design, this time with a focus on incorporating human values in the design process and an emphasis on stakeholder involvement (Winkler & Spiekermann, 2021). VSD uses a tripartite method comprised of a conceptual, empirical, and technical investigation in an iterative process (Winkler & Spiekermann, 2021). The conceptual phase identifies stakeholders and values through literature reviews and brainstorming sessions. Specific attention goes to identifying potential harms and benefits of the technology as well as value tensions (Friedman & Kahn, 2002; Winkler & Spiekermann, 2021). The empirical part uses qualitative, quantitative, or mixed methods approaches to interact with the stakeholders, sometimes extending into co-design approaches. The technical investigation focuses on adjusting the design of the technology to support the identified values and, if possible, to resolve ethical tensions (Winkler & Spiekermann, 2021). This means VSD aligns well with the person-centred and co-design principles discussed above.

The main limitation of this approach is that, because VSD is built on stakeholder input to identify ethical values and tensions, it is not well-equipped to distinguish defensible ethical values from user preferences (Jacobs & Huldtgren, 2021; Winkler & Spiekermann, 2021). Furthermore, VSD has been criticized for not committing to a particular ethical theory, risking the use of a set of values that is unprincipled or unbounded (Jacobs & Huldtgren, 2021). It has been suggested that a mid-level ethical theory, consisting of a cluster of pivotal principles to be used as general guidelines, would be the best fit for VSD, to avoid the risk of naturalistic fallacy (i.e., conflating facts and values) in a top-down approach as well as the lack of clear norms in a bottom-up, casuistic approach (Jacobs & Huldtgren, 2021). The bioethics principles or principlism, focusing on autonomy, non-maleficence, beneficence, and justice, would be one example of such a midlevel ethical theory (Beauchamp & Childress, 2013). However, it has previously been demonstrated that existing bioethical frameworks, such as principlism, fall short in considering the complex challenges presented by DAT and other emerging technologies (Beauchamp & Childress, 2013; Schicktanz & Schweda, 2021). Similarly, a human rights framework has been suggested to provide common guidance and to embed VSD in political and social contexts, but this still risks excluding relevant values or approaches such as care ethics and sustainability (Bleher & Braun, 2023). VSD also does not clearly answer who will be responsible for the quality of the ethical analysis. This could be a bioethicist, but if so, how can the collaboration between them and the technology designers be construed? In brief, VSD offers a person-centred, inclusive approach with end-user-focused co-design, but falls short of integrating the collaboration with bioethicists and may not be well-suited for in-depth ethical analysis.

### Embedded Ethics

Embedded ethics is one of the newer approaches to integrating ethical considerations into the design process, mainly used in AI, robotics, and neuroscience (McLennan et al., 2022; Tigard et al., 2023; Tubig et al., 2024). Embedded ethics can refer to the integration of ethics modules in engineering courses, providing a virtue ethics approach to raising ethical awareness in developers and engineers (Bleher & Braun, 2023). It can also describe the integration of ethics experts into technology engineering teams, meaning ethical, legal, and social issues are considered throughout the developmental process in a collaborative and interdisciplinary way (McLennan et al., 2020, 2022). The latter is of interest to our analysis. Such interdisciplinarity is not new: the ethical, legal, and social implications (ELSI) program of the Human Genome Project is a good example of an earlier approach to socio-technical integration (Tigard et al., 2023). Similarly, the Center for Neurotechnology incorporated a dedicated ethics team or ethics ‘thrust’ into their research activities for ten years (Tubig et al., 2024). These experiences created a new ideal standard for embedded ethics: to have an ethicist or a dedicated team onboard as members of the development team (McLennan et al., 2022; Tigard et al., 2023; Tubig et al., 2024). It implies collaborative work between ethicists and developers to address ethical considerations in an iterative process (McLennan et al., 2020). Although embedded ethics presents a useful approach to collaboration between technology designers and ethicists, it does not necessarily guarantee a person-centred, co-design approach where stakeholders are involved in the technology design. Rather, the focus is on the relationship between technology developers and ethicists, with regular exchanges and active participation in meetings, providing suggestions for stakeholder involvement methods in a toolbox approach but stopping short of requiring them (Willem et al., 2025). Embedded ethics researchers also acknowledge the unresolved risks of tokenism and conflict of interest (McLennan et al., 2022), as discussed above under ETA. How will these ethicists be remunerated (McLennan et al., 2020, 2022)? How will their work be evaluated (Tubig et al., 2024)? What happens when ethical recommendations succumb to power structures driven by the logic of profit orientation (Bleher & Braun, 2023)? Although recommendations have been made for how ethicists may adapt to fit into a team of developers (Tigard et al., 2023), it is less clear how technology development processes can be adapted to incorporate ethicists. Moreover, it is not clear what values and principles should guide the work of these embedded ethicists, echoing a similar critique as heard with VSD (Bleher & Braun, 2023).

To summarize, technological innovation to support aging-in-place should lean on person-centred care and co-design principles to ensure ethical practices. There has been a growing awareness of the need for interdisciplinary collaborative processes that include older adults, carers, (bio)ethicists, and developers in the design and development of DAT to address ethical challenges (Mannheim et al., 2019; Street et al., 2022; Zhu et al., 2022). Whilst several approaches to ethical technology design have been proposed, each method presents strengths and weaknesses. Current methods either lack *stakeholder involvement*, lack adherence to a *normative framework*, do not incorporate *collaboration* between bioethicists and technology designers, or lack attention to *ethical conflicts and tensions* that may arise in messy real-life situations. Our scenario shows that such ethical conflicts and tensions require a thorough understanding of the context of DAT use. Based on this analysis, we propose a pathway for integrating ethics or PIE approach that builds on person-centred care and co-design principles to incorporate inclusivity, flexibility, collaboration, and practicality. PIE builds on these three existing methods, incorporating their strengths and aiming to overcome their limitations, while also presenting a clear process to operationalize more conceptual or theoretical ideas. In doing this, it seeks to answer the ‘how’ question (Morley et al., 2020) and contribute to a practical ethical framework for incorporating ethics into the design process, while setting specific requirements about what this process should entail.

## Pathway for Integrating Ethics

Our central question is: how can we integrate ethics into the design of DAT applications guided by person-centred care and co-production principles? More specifically, how do we identify and resolve ethical tensions and dilemmas, or at least manage them in the DAT design?

We argue that this process should on four general values derived from *person-centred care* and *co-design* principles.

1. **Inclusivity** is the commitment to treating all individuals with respect, valuing differences, and encouraging diversity of stakeholder perspectives, to ensure decision-making is informed by comprehensive information and to mitigate potential biases (Gerrard et al., 2025; Goodwin et al., 2025; Meskó & de Bronkart, 2022; Sanz et al., 2021). This also means that, in the co-design process, vulnerable stakeholders are not ignored;
2. **Flexibility** is the willingness to modify or compromise, including the ability to adapt to unforeseen challenges, adjust to changing circumstances, and show resilience when navigating dynamic environments (Tang et al., 2024). Flexibility is required at the level of the DAT, as it needs to serve different end-users, avoiding a one-size-fits-all approach (or, help design adaptable and customisable technology) (Pilosof et al., 2023), but also at the level of the PIE approach itself so that it can be applied to different types of DAT and other technologies;
3. **Collaboration** is the process of working together towards a common goal by leveraging the diverse skills, perspectives, and ideas of participants. It is the act of creating or producing something together, recognising that each individual’s contributions are essential to the success of the whole. This includes stakeholder engagement as well as bioethicist involvement. The role of ethicists needs to be fleshed out, to find a balance between embedding ethicists in the design process while also safeguarding their independence to avoid conflicts of interest and ‘ethics washing’ (Hao, 2019);
4. **Practicality**: Practicality is being realistic and effective in achieving intended results with the available resources and constraints. It means being grounded in real-world conditions and focusing on the functionality, usability, and straightforwardness of solutions or tools. At the same time, imagination and improvisation play a central role in the design process (Rylander Eklund et al., 2022). Attention should be given to bridging the gap between theory and practice, acknowledging that adjustments may be required due to the ‘messiness’ of real-life circumstances and ensuring recommendations are useful for designers and users alike.

To meet these criteria, we propose a 4-step iterative process resulting in the integration of ethics into technology design. This process does not require existing design processes to be overhauled; rather, it can be seen as an augmentation or extension of current design methods to include ethical considerations. In this sense, it is a design methodology-agnostic, iterative approach that aims to be flexible, while offering support to navigate the nuances and complexities of both clinical and ethical considerations in the DAT design space. However, integrating ethics into technology design will be more efficient if technology designers have had some prior training in ethics, and ethicists have had some prior training in digital health technology. Ethics courses for designers exist, but the range and depth of exposure of design students to ethics is still limited. Similarly, not many philosophy or bioethics students receive formal training in digital health technologies. Starting the design process with designers attuned to ethics and ethicists educated in digital health will make a close and effective *collaboration* and mutual understanding more likely. Moreover, it will help achieve *flexibility* in thinking and improve the capacity to consider problems from multiple perspectives and to adjust to challenges. Lastly, the PIE has been developed as a tool to integrate ethical considerations in a design process that first identifies an actual need or problem. Although it seems self-evident, design often still starts with a technological solution in need of a problem. We believe applying design thinking principles, focused on understanding the problem or need and then tailoring specific solutions to these identified needs, is best suited to PIE integration and leads to better technological outcomes. However, we have developed our pathway keeping other design methods in mind as well, so it could be integrated into a design process that starts from an existing DAT. The four steps of the PIE are discussed below.

### Step 1: Bringing Ethicists and Designers Together

Previous approaches to embedded ethics have mainly seen ethicists join a team of developers to support the ethical considerations of the project and to provide teaching via workshops (Tigard et al., 2023). However, as discussed above, solely identifying ethical principles is not sufficient. Rather, ethical tensions need to be explored, and at the end of this process, ethicists should be able to provide the designers with *practical* recommendations that can guide design toward the management of these tensions.

In the first step of the process, we assume the designers are either exploring a need and clarifying requirements and limitations, or are developing a project plan (Wang et al., 2024a). This preparation phase typically involves clients, designers, and occasionally other stakeholders, such as hospitals. Ideally, it would also include ethicists and users to determine the project requirements, but there is ongoing debate around at what point and to what extent end-users should be involved in the design process (Wang et al., 2024a). Here, early ethicist involvement could be highly valuable in setting appropriate and ethically sound goals.

We propose to establish a *collaborative* partnership that preserves the ethicists’ independence, minimises conflicts of interest, and avoids tokenism or ‘ethics washing’. This can be realised in a few different ways. Ideally, the ethicists working on a project are not directly employed or remunerated by the design team or industry partner. In this case, remuneration for the bioethicists’ work could be set up via a central body, institution, or grant, avoiding a conflict of interest in this sense. Alternatively, ethicists could be working as consultants remunerated by the industry partner if a clear contract has been drawn up to ensure they can work independently. This may be accomplished, for example, by publishing a publicly available report of the ethicists’ findings and recommendations, while protecting the designers’ intellectual property, similar to other academic-industry partnerships (Goodson et al., 2025; Pantanowitz et al., 2022). Ethicists will need a clear understanding of the technology to move through the next steps, meaning there will be ongoing interactions with the developers as they progress. In other words, this will be an iterative *collaborative* process, particularly given there may be more than one solution to the problem. At the end of this step, ethicists and designers should reach an agreement about the terms, aims, conditions, and scope of their collaboration and have set out the specific process they will follow to integrate ethics into their design project.

### Step 2: Identifying Ethical Tensions

In the second step, ethicists would explore the ethical values, principles, and tensions relevant to the DAT solution that the design team are working on. Again, this could be at the stage of problem identification, clarifying user needs, or there could be an early idea or prototype for a technological solution. While the designers are working through these steps, ethicists could use two methods to analyse arising ethical tensions in-depth. A *literature review* is the first part of the process of identifying values and tensions. Previous work has identified relevant values for Big Data research, ethical tensions that occur with smart home technology for frail older adults aging in place, and there are numerous frameworks identifying values and principles for AI use (Jobin et al., 2019; Wang et al., 2023; Xafis et al., 2019). The literature review should adhere to a clear structure and will likely be scoping or systematic (Munn et al., 2018; Peters et al., 2021; Tricco et al., 2018). Any literature review should be contextualized, i.e., adapted to and understood in the local context, taking into account local cultural and social aspects, including thinking about potentially vulnerable stakeholders, non-users, indigenous peoples, and other potentially underrepresented groups (Kiran et al., 2015; van de Poel, 2016). The second part of this step should involve *interaction with a small group of stakeholders* to understand the challenges and decide on priorities. This would include the ongoing collaboration between ethicists and designers, but also bring in other relevant stakeholders such as healthcare providers, informal caregivers, older adults, etc. This group of stakeholders may be the same individuals the designers interact with throughout their process. This would lay the foundation for person-centred care and co-design in the next steps. The research should not solely focus on values and principles identified in the literature review: stakeholders should be able to bring new questions or insights to the table. This also means that attention needs to go to prioritization or the identification of the most important ethical tensions based on the literature review and stakeholder engagement process (Kabacińska et al., 2023). Overall, ethicists should also clearly specify which normative framework will guide their work (Jacobs & Huldtgren, 2021). This framework should be relevant to the DAT context, and it should be made transparent how this framework will be applied throughout the PIE process.

Throughout this step, ongoing *collaboration* with the designers is ensured, as is *inclusivity* through the early involvement of a small group of stakeholders, including end-users. At the end of this step, ethicists should have created a concise overview of the most relevant values and principles, the tensions that arise within them, and situated examples of these tensions in the form of cases and/or vignettes. This would be very similar to the illustrative scenario we have described above, albeit in a more extensive form, including early reporting to the design team, raising ethical concerns and considerations that may influence the choice and subsequent design of the DAT solution, and also materials that can be used for stakeholder engagement in step 3.

### Step 3: Exploring Solutions to Ethical Tensions Through Stakeholder Engagement

The cases, vignettes, and other material developed in the second step can now be presented to a larger group of relevant stakeholders aimed at finding solutions to the identified ethical tensions. As discussed above, stakeholder selection is a challenging process. *Inclusivity* requires the involvement of people from different backgrounds, including non-users and people who may decline or oppose DAT use. As a general guideline, we recommend meaningful interactions with older adults themselves, their family members and carers, healthcare professionals (in particular those involved in home care), and organizations representing older adults. Specific attention should go to otherwise underrepresented groups where the DAT may have a different impact, e.g., people living in poverty, facing abuse, living with disabilities, people from ethnic minorities and Indigenous people. Stakeholder engagement can build on both quantitative and qualitative methods, but we would deem some qualitative methods essential to elicit responses to the previously identified ethical tensions (Robinson et al., 2022). Eliciting techniques may involve the use of scenarios, envisioning cards, or similar methods: some participants may not have any experience with DAT use, and eliciting techniques can make the conversation more meaningful (Berridge et al., 2020; Robinson et al., 2022). Interviews and focus groups can be used in the early design phases. In later phases, prototypes can be used in stakeholder engagement, presenting the DAT to the stakeholders in interviews or focus groups, or participant observation, once the DAT is ready for implementation (Smits et al., 2022). In this phase, tensions between values or between stakeholders are brought out more explicitly. For example, previous research has shown that children and older adults often disagree on the acceptability of monitoring with regard to privacy, with the use of cameras proving particularly divisive (Berridge et al., 2020). Here, the presentation of alternative technological solutions and their acceptability may be a useful tool to explore this ethical tension more in-depth, by discussing alternatives to camera use, restricted access or sharing options, and the possible use of AI for data interpretation. This means the ethicists would then focus on potential solutions to the ethical tensions, and their strengths and weaknesses. The outcome of this step is to select the most suitable solutions to ethical tensions from an end-user perspective. This is where *flexibility* should be emphasised: rather than finding the perfect or a one-size-fits-all solution as the outcome of the stakeholder engagement, clear suggestions on how the DAT needs to be modifiable and customisable to alleviate ethical tensions should be brought forward by the ethicists’ work. Finally, stakeholders are also encouraged to identify any *practical* limitations of the proposed DAT in terms of functionality and usability in real-world settings.

### Step 4: Integrating the Identified Solutions into the Technology Design

Once the proposed solutions to ethical tensions have been identified, they need to be integrated into the technology design process which will allow designers to build a prototype focused on *practicality*. Again, ongoing collaboration between ethicists and designers is a key part of this step. There will undoubtedly be trade-offs, for example, due to technical, cost, and other resource-related limitations: involving ethicists in the decision-making process can help with prioritizing system requirements. This step can be integrated into existing types of prototype development techniques. This means designers or industry partners can adhere to their chosen development methodology but integrate ethical considerations as they design deliverables. These deliverables can then be taken up into an iterative process involving re-engagement with ethicists and stakeholders. From an ethical perspective, this would mean continuous collaboration with the design team during the initial product development, and re-engagement for subsequent product updates. At this point, an evaluation of the PIE process is also appropriate. Measuring the ethics integration effectiveness is challenging (Tubig et al., 2024); however, it could be done through a mixed methods approach in which evaluation criteria are applied to the process and output, i.e., technology.

**Fig. 1:**
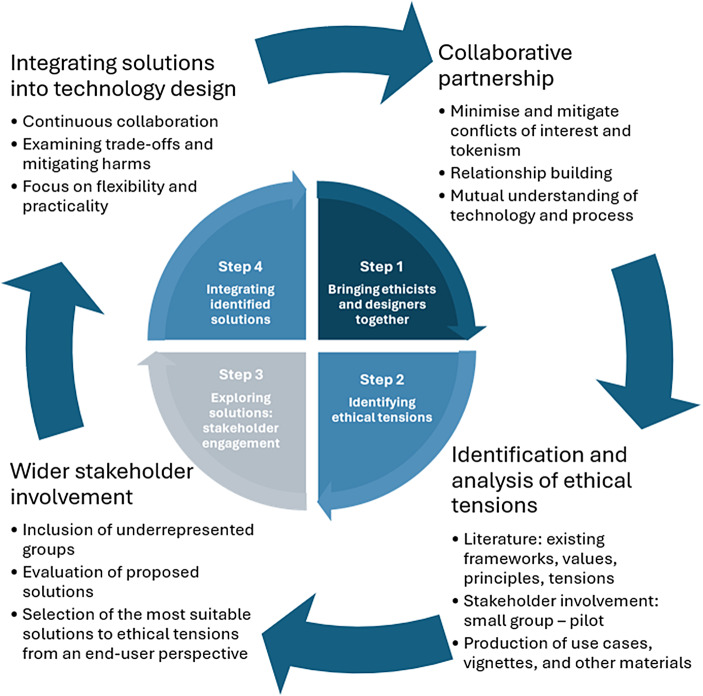
Visual representation of the PIE process

The outcome of the method is a *person-centred* technology that has been developed in close interaction with its *diverse* stakeholders in a *co-design* process. This is expected to lead to more *flexible* technology as it needs to cater to varied needs and also changing needs and attitudes toward technology throughout users’ lives (Bechtold et al., 2024; Elers et al., 2018) (Fig. 1).

## Discussion

The PIE approach provides a practical framework for integrating ethics into health technology design, with a strong emphasis on person-centred care and co-design. It builds on three existing methods for ethical design: ETA, VSD, and embedded ethics, incorporating their strengths and aiming to overcome their limitations. At the same time, its novelty lies in presenting a clear process that operationalises ethical values and principles into technology design in a design-methodology-agnostic approach. By creating a practical process in four steps, PIE seeks to move from highly abstract principles to their application, bringing about actual change in the design of these technologies (Morley et al., 2020). Although PIE overcomes several challenges and offers a practical way forward in terms of integrating ethics, it grapples with ongoing issues and generates new questions. We will address five points here: 1/ how PIE overcomes the limitations of the methods it builds on; 2/ the ‘chicken-and-egg’ problem, or how early bioethicists should be involved in the design process; 3/ the ongoing risk of tokenism and how to organize remuneration; 4/ the challenge of evaluation of the PIE process, or how to measure the impact and define success; and 5/ how to ensure that co-design and the involvement of stakeholders is meaningful.

First, in our analysis of the three existing methods, we identified several potential issues: a lack of stakeholder involvement, inadequate adherence to a normative framework, insufficient collaboration between ethicists and designers, and insufficient attention to ethical conflicts and tensions that may arise in complex, real-life situations. The PIE approach places stakeholder involvement at the centre of its methodology, builds on the collaboration between ethicists and designers in its initial phase, and uses case studies to focus on ethical tensions in its second and third phases, therefore addressing these concerns. It does not explicitly adhere to one ethical framework; however, it does require ethicists to specify which normative framework will guide their work (Jacobs & Huldtgren, 2021). An ongoing point of contention is whether transparency in the normative framework is sufficient or whether a more robust approach, including justification, contextualisation, and evaluation, is warranted. Overall, it outlines several requirements ethicists need to meet to ensure a thorough consideration of ethical issues.

Second, the ‘chicken-and-egg’ problem remains difficult to answer: how early should bioethicists be involved in the design process? This is very similar to the question of how early stakeholders, more broadly, and end-users, more specifically, should be involved, and the overall recommendation is to involve stakeholders actively in all stages of the project (Wang et al., 2024a). If true co-design is applied, technology design begins with stakeholder involvement to identify actual needs and ensure that any new technology idea meets user requirements. Similarly, we argue that involving ethicists from early on, ideally in the idea and project generation phase, could provide value to the resulting technology. It may help avoid misalignment, such as seen in the reluctance of many older adults to wear neck pendants. Nevertheless, the PIE process may also begin later, when a clear idea for a technology is already on the table. Here, the question will be whether the ethicists can suggest fundamental changes to the idea or plan based on their early findings.

This leads us to the ongoing risk of tokenism: despite the practical PIE approach, designers may choose to ignore the findings and recommendations of the involved ethicists, but still claim the resulting technology is ‘ethical’ because they had ethicists on board. Ensuring that ethicists can maintain a level of independence and, for example, publish their findings and recommendations, could at least partially solve this issue. This would need to take into account any intellectual property restrictions. A separate source of remuneration could also contribute to this, although we are aware that our suggestion to establish remuneration through a central body would require considerable time and organizational effort. This could be incorporated into regulatory processes, when they are put in place at a local or national level, to provide a long-term solution.

Fourth, how do we measure the impact of this PIE process and define success? Experience from a research project embedding an ethics team suggests applying productivity metrics from scientific practice to ethics-related work, such as grants, publications, and career trajectories (Tubig et al., 2024), but this may not be applicable to technology design, and it does not evaluate the actual outcome, i.e., how ethically sound the technology is. Our suggestion is to use a mixed-methods approach and create evaluation criteria. Further thought should go to who will conduct this evaluation and how the results can be disseminated. Lastly, we have tried to specify certain requirements for the stakeholder involvement, aiming to ensure meaningful and authentic co-design where stakeholders play an active role (Gerrard et al., 2025; Goodwin et al., 2025; Sanz et al., 2021). Despite these attempts, a risk of tokenistic stakeholder involvement remains, and the quality of this involvement is difficult to assess. We suggest that developing evaluation criteria for the PIE process would be a logical next step.

## Conclusion

In this paper, we have proposed a novel approach for designing DATs that centres on the principles of patient-centred care and co-design: a pathway for integrating ethics (PIE). This approach comprises four stages that embody four fundamental values: inclusivity, collaboration, flexibility, and practicality.

The PIE approach aims to enable (1) a *close collaboration* between the design team and ethicists throughout the project, within clearly defined terms and conditions, (2) a strong emphasis on *stakeholder involvement* including end-users, and (3) a focus on *identifying and resolving ethical tensions* that may arise with the use of digital technologies, in this case, DAT for older adults. Its application should result in a more responsive and robust end-product that better meets the needs of older adults and supports aging-in-place, combining the benefits of co-design and person-centred care. We have focused on the context of care for older adults and digital assistive technologies to demonstrate how this new method could work, as the approach requires an understanding of the context and perspectives of the involved stakeholders. However, we intend for the PIE approach to be applicable to other contexts. Further research can focus on applying the PIE approach to specific projects and validating this approach in different technological contexts.

## Data Availability

Not applicable.
